# Hays of Novel-Improved Legume Cultivars: Phytochemical Content and Nutritional Value

**DOI:** 10.3390/plants13213058

**Published:** 2024-10-31

**Authors:** Eleni D. Myrtsi, Dimitrios N. Vlachostergios, Christos Petsoulas, Epameinondas Evergetis, Sofia D. Koulocheri, Serkos A. Haroutounian

**Affiliations:** 1 Laboratory of Nutritional Physiology and Feeding, Department of Animal Science, School of Animal Bioscience, Agricultural University of Athens, Iera Odos 75, 11855 Athens, Greece; elenamirtsi@aua.gr (E.D.M.); epaev@aua.gr (E.E.); skoul@aua.gr (S.D.K.); 2 Institute of Industrial and Forage Crops, Hellenic Agricultural Organization ELGO-DIMITRA, 41335 Larissa, Greece; petsoulaschristos@elgo.gr

**Keywords:** fabaceae, legumes, hay, cicer, lathyrus, medicago, phaseolus, pisum, vicia, polyphenols

## Abstract

The imperative need to produce safe foodstuffs using environmentally sustainable practices has highlighted the incorporation of legumes in human and animal diets as an emerging nutritional staple. Since legumes comprise a family of plants known to display an extensive agricultural diversity with significant bioactivities, we report herein the exploitation outcome of the nutritional and bio-functional content of hay, derived from the post-harvest aerial parts of eight novel-improved Greek cultivars belonging to the following six taxa: *Larthyrus sativus* L., *Medicago sativa* L., *Cicer arietinum* L., *Pisum sativum* L., *Vicia faba* L., and *Phaseolus vulgaris* L. In specific, the phytochemical content of their hay extracts was screened against the presence of 37 fatty acids and 67 polyphenols using, respectively, GC-FID and LC-MS/MS instruments, while the determination of their total phenolic and tannin contents was achieved with spectrophotometric methods. In this respect, the presence of 26 different fatty acids was detected of which the acids linoleic, linolenic and palmitic were traced in all extracts in concentrations exceeding the 10 mg/g, while the oleic acid was determined as the major component of *C. arietinum* (77.58 mg/g), *V. faba* (36.11 mg/g), and *L. sativus* (12.89 mg/g) extracts. In addition, 30 different phenolics were traced as extracts’ components with the most abundant the molecule of isoquercetin, which was present in six extracts and isoliquiritigenin, calycosin, and chlorogenic acid which were detected in five extracts. Finally, the antioxidant properties of the extracts were evaluated by performing both DPPH^•^ and FRAP assays, which highlighted the *V. faba* extract as the most potent in both tests, followed by the extracts of *P. sativum* and *P. vulgaris*. Results herein are indicative of the significant advances achieved, for the improvement of investigated plant cultivars with respect to their utilization as nutritional crops, since these novel cultivars hays have been found to contain significant amounts of essential nutrients in the form of fatty acids along with bioactive ingredients in the form of polyphenols, while simultaneously they were determined as deprived of tannins that constitute essential anti-nutritional factors.

## 1. Introduction

The production of plant-originated food, feed or industrial raw materials generates significant amounts of biomass as the main byproduct of the respective plants’ cultivation. Inherently, this biomass is transformed into hay in order to be incorporated into integrated crop-livestock systems feeding. Recently, this low-added-value procedure has attracted considerable research interest towards the optimization of applied management practices aiming to produce a higher-quality feed [1,2,3,4,5,6]. Among the various plant species exploited for this purpose, legumes hold a prominent position since their sustainable character can provide numerous environmental benefits [4,7,8]. Legumes are classified into Fabaceae family plants and comprise a group of modern-day crops that closely follow cereals with respect to their total cultivation area. The latter are well known for their global expansion which was augmented by 152.6% (128.37 m ha) during the last 50 years, while in the same period, their production volume was increased by 548.6% [7]. 

Previous research results have concluded that legumes can play an important role in sustainability plans, since they may be implicated in multiple functions. Specifically, legumes are incorporated into both human and animal diets providing valuable nutrients that benefit their health. Simultaneously, they play a pivotal role in the environment by increasing soil fertility through nitrogen fixation and contributing to climate change mitigation [8,9,10]. On the other hand, the broad diversity of the Fabaceae family plants that exceed the 19,000 taxa, each representing numerous cultivars [10], comprises a serious drawback which undermines the development of widely applicable post-harvest management solutions. Thus, literature abounds with numerous fragmented endeavors for the development of processes for their efficient management with outstanding examples of their utilization as organic fertilizer [11], as forage and/or silage [12], and as a source of bioactive phytochemicals [13]. This last feature has been determined as a promising and environmentally sound alternative for the manipulation of legume biomass [13] since these plants are established as sources of protein, carbohydrates, fat, and vitamins [14]. In this respect, the phytochemical content of legume hays in bioactive natural products is closely connected with livestock growth and welfare, since their nutritional value is closely connected to their biochemical content [10,15]. 

Among the numerous phytochemicals contained in legume tissues, fatty acids comprise a distinctive structural cluster of nutrients. Fatty acids are classified into primary metabolites and comprise crucial components of lipids metabolism. Although their anti-inflammatory, anticancer and antiatherogenic properties have been extensively studied [15,16,17], there are only limited reports on the assessment of the individual fatty acids presence in legume tissues, since most studies are focused on their total fatty acid content [15].

Besides the determination of legumes’ primary metabolite content, recently the detection/isolation of various secondary metabolites from their plant tissues has also attracted considerable research attention as a compelling subject. This interest is inspired by the potency of secondary metabolites for incorporation into various products, such as foods [18], feed [19], pharmaceuticals [20], and cosmetics [21]. Among the numerous groups of natural products classified into plants’ secondary metabolites, polyphenols comprise the most extensively studied class of molecules, with phenolic acids and flavonoids being the most pronounced structural cluster as a consequence of their numerous well documented bioactivities [22,23], including the antioxidant, anticancer and antibacterial properties [15]. On the contrary, tannins comprise a class of bioactive molecules with well-established anti-nutritional properties that affect feed’s palatability [24,25]. It must be noted, however, that there is a scarcity of available reports enabling the connection of different legume cultivars’ molecular diversity with the respected bioactivities [26]. 

The main objective of the present endeavor is the investigation of eight legume hays produced from novel-experimental Fabaceae family cultivars, with respect to their phytochemical content and bioactivity. For this purpose, we have employed a phytochemical diversity study approach that includes hays’ sequential extraction and determination of their total phenolic and tannin contents, as well as the fingerprinting of their fatty acids and phenolic compounds. These data were elaborated with the results of their antioxidant capacities assessments. The final goal is to determine those displaying the best characteristics for use in livestock feeding and to discuss the biochemical functions of each *taxon* through the comparison of the herein presented results with previous reports, enabling us to extract conclusions concerning the influence of specific cultivars on each *taxon’s* phytochemical content. 

## 2. Results

Research samples were obtained by the fractionated extraction of the respective plant’s aerial parts, using consecutively the following solvents: hexane, dichloromethane, and methanol. Hexane extracts were utilized for the determination of fatty acids content and the methanolic extracts for the assessment of their phenolic compounds content. The antioxidant capacity assessments were performed on all extracts. The abbreviated names of samples along with their extraction yields are presented in Table 1. 

### 2.1. Determination of Total Phenolic and Tannin Contents

The total phenolic (TPC) and tannin (TTC) contents of the methanolic extracts were determined spectrophotometrically, indicating that the TPC values of most extracts are ranging between 10 and 20 mg of Gallic Acid Equivalents (GAE) per g of extract, with the notable exception, the fava VFP extract, which contained 46.9 mg GAE/g. On the other hand, the presence of tannins was verified in only two samples, namely VFP fava and PSO pea extracts which exhibited respectively TCC values of 7.7 and 25.3 mg of Catechin Equivalents (CE) per g of extract. The detailed results are included in Table 2.

### 2.2. Antioxidant Properties Assessment

The antioxidant properties of investigated extracts were evaluated by performing two assays for the determination of their free radical scavenging (DPPH^•^) and reducing (FRAP) capacities. The DPPH^•^ bioassay results indicated as most active the methanolic extracts and as non-actives the dichloromethane extracts, while FRAP bioassay provided consistent results for all extracts, with the dichloromethane extracts being the most active. The detailed results are presented in Table 3. 

### 2.3. Fatty Acids Fingerprinting Results

**Figure 1:**
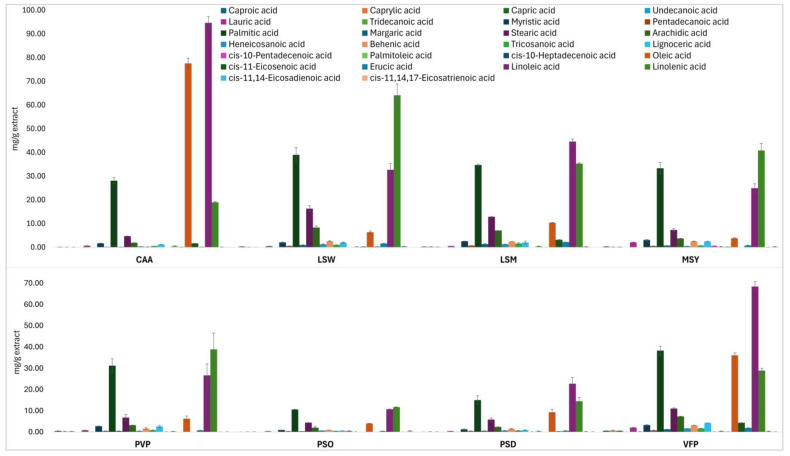
Fatty acids content of the investigated Fabaceae plants’ hexane extracts.

In total, the presence of 26 fatty acids was revealed in all hexane extracts. Among them, 16 compounds are characterized as saturated fatty acids (SFA), six mono-unsaturated fatty acids (MUFA) and four poly-unsaturated fatty acids (PUFA). The total volume of fatty acids in the investigated extracts ranged from 234.5 mg/g for chickpea CAA biomass to 49.22 mg/g for pea PSO biomass. The respective cumulative results are presented in Figure 1, while the detailed quantitative content of individual fatty acids (as mg/g extract) has been provided in Table S1 of the Supplementary Materials section.

### 2.4. Results of Phenolic Molecules Fingerprinting 

In total, the presence of 30 phenolic phytochemicals was determined in the investigated methanolic extracts. The individual concentrations of the phenolic compounds detected in the studied biomass extracts are presented in Table 4. Chickpea CAA biomass extract was highlighted as the richer since it was determined to contain 21 polyphenols, while the chemical diversities of kidney PVP and fava VFP biomass extracts were also particularly rich in polyphenols as they are found to contain 15 and 16 molecules respectively. The individual concentrations of the phenolic compounds detected in the studied biomass extracts are presented in Table 4.

### 2.5. Screening of Fabaceae Family Crops Utilizing Hierarchical Clustering Analysis (HCA) and Principal Component Analysis (PCA)

#### 2.5.1. Hierarchical Cluster Analysis (HCA)

A hierarchical clustering analysis (HCA) was conducted to classify the extracts of the eight investigated legume cultivars using the “complete linkage” method. This approach merges clusters based on the maximum distance between any two points in the clusters, considering a total of 30 traits related to phytochemical components and antioxidant properties.

**Figure 2:**
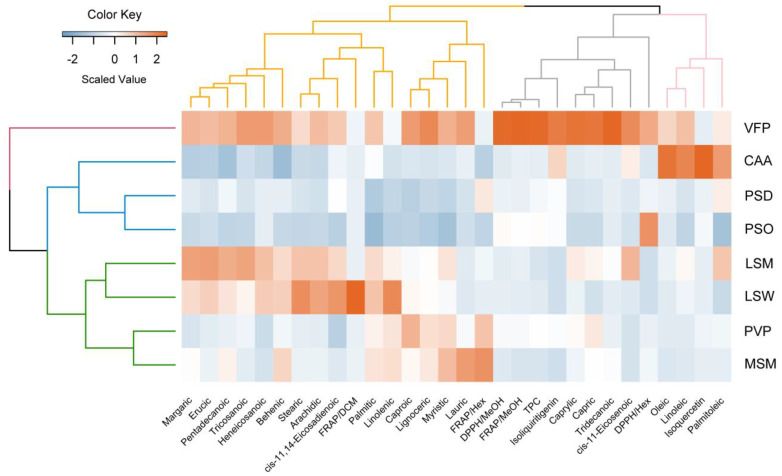
A heatmap was generated for each of the eight legume cultivars methanolic extracts, depicting 30 characteristics related to phytochemicals and antioxidant properties. The heatmap utilized a color-coded scale where orange indicated an increase and blue represented a reduction in scaled values across these characteristics. To explore the relationships among the descriptors and cultivars, hierarchical cluster analysis was applied using the complete linkage method. The resulting dendrograms visually illustrate how these descriptors and cultivars are clustered based on their similarities in the observed characteristics.

Based on the HCA results, the eight cultivars were grouped into three distinct clusters (see Figure 2). The first cluster includes cultivar VFP, while the second cluster is comprised of cultivars PSO, PSD, and CAA. The third cluster consisted of cultivars LSM, LSW, PVP, and MSY.

Furthermore, a heatmap was generated to visualize the values of these 30 traits, emphasizing the notable performance of the VFP variety across the investigated traits. This effect is directly associated with Table 2 results indicating that the VFP methanolic extract prevails with respect to the TPC value. For a detailed understanding of the correlations between their chemical properties, refer to Figure S1 in the Supplementary Materials section.

#### 2.5.2. Principal Component Analysis (PCA)

**Figure 3:**
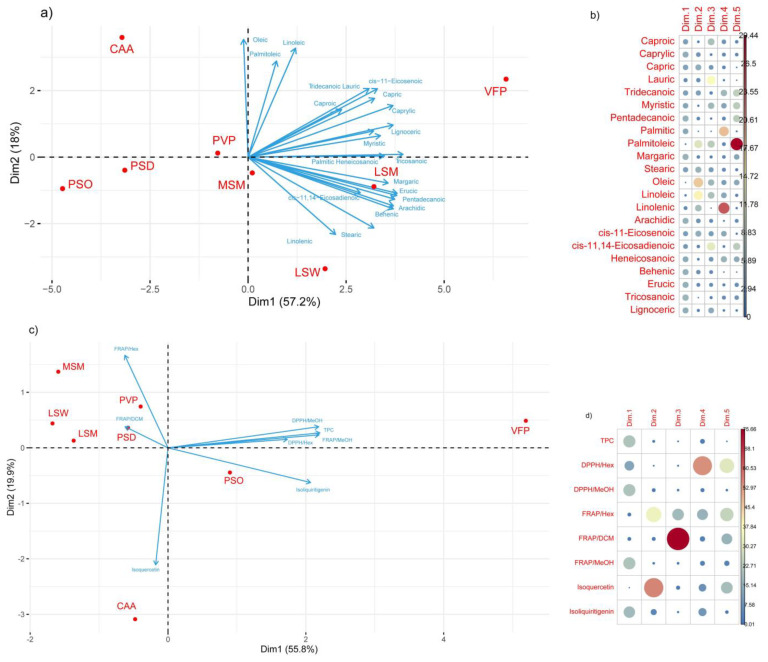
PCA results visualization. (**a**) This biplot illustrates the relationship between legume cultivars byproducts and their fatty acid composition. The arrows represent the fatty acids, while the red points represent the Legume cultivars. The length and direction of the arrows indicate the correlation between each fatty acid to the first five principal components. (**b**) This correlation plot shows the contribution of each fatty acid to the first five principal components. The color scale and size of the points indicate their respective contributions. (**c**) In this biplot, Legume cultivars are depicted along with their antioxidant properties. Similarly, the arrows represent different antioxidant properties, while the red points represent the Legume cultivars. The length and direction of the arrows indicate the correlation between each antioxidant property and the principal components. (**d**) This correlation plot shows the contribution of each antioxidant trait to the first five principal components. The color scale and size of the points indicate their respective contributions.

PCA is a statistical method used in an unsupervised manner to uncover underlying patterns within a dataset, revealing hidden similarities and/or differences. Specifically, PCA was employed to identify correlations between the compositions of phytochemicals and antioxidant properties across various species and to categorize them. Two distinct analyses were conducted: one focusing on fatty acids composition and the other incorporating polyphenol content alongside antioxidant properties (see Figure 3).

In the first PCA analysis, approximately 57.2% and 18% of the total variance were explained by the first (Dim1) and second (Dim2) principal components, respectively, cumulatively accounting for 75.2% of the total variance. Notably, fatty acids such as Arachidic, Caprylic, Behenic, Pentadecanoic, and Margaric made significant contributions to these principal components, with others closely following. Regarding legume cultivars, the analysis revealed strong clustering among varieties of the same species, particularly grouping two Pisum sativum varieties (PSO and PSD) and two Lathyrus sativus varieties (LSM and LSW) together. Additionally, VFP exhibited the highest contribution to the first two components.

In the second PCA analysis, the first (Dim1) and second (Dim2) principal components explained 55.8% and 19.9% of the total variance, respectively, collectively elucidating 75.7% of the total variance. Dim1 showed a strong positive correlation with antioxidant capacity measured by FRAP/MeOH and DPPH/MeOH assays, total phenolic content (TPC), and isoliquiritegenin. Dim2 exhibited notable correlations with isoquercetin and the FRAP (Hex) assay. This analysis highlighted a strong clustering between LSM and LSW, with moderate clustering observed for PSO and PSD. However, similar to the first PCA, a significant variability was observed among species within the same genera. Once again, VFP demonstrated the highest contribution to the first two components.

## 3. Discussion

The scientific evaluation of the herein presented results can somehow be considered as a two-fold endeavor since it includes two distinct approaches. The first focuses on the extracts of chickpea (CAA), kidney bean (PVP), fava (VFP), and the two pea (PSO and PSD) cultivars and relates the herein obtained results with the relevant data determined for their respective bean extracts [26]. The second approach involves all extracts investigated and relates the respective results with previous literature findings, which although numerous, present crucial gaps with respect to the origin and the level of the herein applied analyses. It must be noted that the lack of a quality control specimen, not foreseen in the initial agronomical experiments, can be considered as a limitation for the discussion of the herein obtained results. This drawback can be rationalized considering that hays used for livestock feeding originated from a broad variety of plants with diverse phytochemical contents, making difficult their reliable comparison with only one specific hay. Nevertheless, since the initial content of various hays has already been studied previously, we have incorporated in our discussion the respective literature data. Finally, it must also be noted that since the main target of this endeavor is the exploitation of the *supra taxa* dimension of the novel cultivars, our efforts were mainly focused on the study of the relevant phytochemical content of the pulses from the respective time crop. 

### 3.1. Total Phenolic and Tannin Content

#### 3.1.1. Total Phenolics

The measured TPC values for the aerial parts of the investigated herein eight legumes indicated that seven cultivars display comparable activities with values ranging from 11.1 to 19.5 mg GAE/g extract while the VFP cultivar excelled displaying a value of 46.9 mg GAE per g of extract. Since five of these extracts have originated from herbal material that also produces edible beans, namely CAA, PVP, PSO, PSD, and VFP cultivars, we compared their TPC values with those determined for their beans [26]. The respective results revealed that the aerial part extracts display TPC values significantly higher as compared to those of bean extracts. Specifically, the TPC value of the CAA chickpea cultivar aerial part extract was 14.2 mg GAE/g while the respective value for its bean extract was 4.2 mg GAE/g; Similarly, the investigated kidney PVP cultivar extract exhibited a TPC value of 18.9, in contrast to its bean extract which was 8.2 mg GAE/g. For the PSD pea cultivar, this variance was somehow lower since the TPC value for its aerial part was 16.7 and for its beans 12.7 mg GAE/g. Finally, it is noteworthy that the VFP fava extract exhibited a higher TPC value (46.9 mg GAE/g), despite that no phenols were traced in its bean extract. The only extract not following this pattern was the PSO pea cultivar which displayed 26.2 mg GAE/g TPC value for its bean extract and 19.5 mg GAE/g extract for its aerial part. Therefore, the present study results reveal that the main phenolic content of legume plants is preferably located in their hay as compared to their beans, with only the exception of the PSO pea cultivar.

The aforementioned results are comparable to those reported by Sibul et al. (2016) [27], a study considered a key reference for the field because it includes the exploitation of the phenolic profile of several samples obtained from the seven most cultivated legume plants. In specific, the herein reported TPC values for pea cultivars ranged respectively from 16.7 to 19.5 mg GAE/g for PSD and PSO extracts, values exceeding the previously reported TPC values which ranged from 0.134 to 0.255 mg/g extract [27]. Similar observation was revealed for the chickpea CAA cultivar, which herein was found to display TPC value of 14.2 mg GAE/g extract, while the reported respective values ranged from 3.094 to 5.177 mg/g extract [27]. In addition, previous research findings on Chilean kidney beans extracts have reported TPC values for six cultivars ranging from 6.48 to 7.01 mg GAE/g extract [28]. These values are almost one-third of the 18.9 mg GAE/g value for the PVP extract measured herein. Finally, the TPC value of VFP faba cultivar beans was measured as 46.9 mg GAE/g extract which is comparable to previously reported values for its leave and twig extracts that displayed TPC values 16.4 and 63.1 mg GAE/g respectively [29]. 

Beside these five legumes extracts used mainly for human nutrition, study herein includes the investigation of the alfalfa MSY extract, a legume plant used exclusively as feed and/or fodder plant. Previous reports exploiting the tissue culture extracts of this variety has reported TPC value 19.34 mg GAE per gram of dry material [30], a value comparable to herein determined value of 11.4 mg GAE per g of extract. 

With respect to the aerial parts of LSW and LSM *Lathyrus* cultivars, both used as human nutrients, there are no relevant previous data for comparison, since they belong to a path less exploited by contemporary research. The TPC values reported herein for these two grass-pea extracts were respectively 11.1 and 13.1 mg GAE/g of extract, values significantly higher to the reported value of 0.156 mg/g extract for similar cultivars [27].

#### 3.1.2. Total Tannins

The tannin content of the eight investigated samples was particularly poor since only the methanolic extracts of PSO pea and VFP fava bean cultivars were found to contain tannins with respective values of 25.3 and 7.7 mg CE/g extract. This pattern of deprived tannin content of the aerial parts of investigated plants is consistent with the corresponding results obtained for their respective beans [26] and constitutes a significant advantage for their incorporation in feeding rations since tannins are well-known antinutritional factors. It is well established that tannins display the ability to bind with several nutritional components, such as proteins, iron, etc., reducing thus their digestibility. 

The most notable results were obtained for the two pea cultivars, which displayed a reverse behavior. In specific, the PVP bean extract exhibited a TTC value of 38.5 mg CE/g, displaying a completely different profile as compared to its aerial parts which did not contain tannins. On the contrary, tannins were not detected in PSO bean extract, while the extract of the aerial part of the plant displayed a TTC of 25.3 mg CE/g extract. Similar behavior was observed for the fava bean VFP cultivar, where the TTC status reversed from absent in bean extract to present at 7.7 mg CE/g in the aerial parts extract. 

### 3.2. Antioxidant Activity Evaluation

#### 3.2.1. DPPH^•^ Assay

The antioxidant properties of the Fabaceae plant’s biomass are relatively underexplored since most the relevant research endeavors have focused on the exploitation of the activities of their seeds. The herein acquired results for the aerial parts extracts of the exploited eight cultivars present a diverse profile with respect to their free radicals scavenging bioassay. As most potent were determined in the methanolic extracts with bioactivity measurements ranging from 0.4 to 84.6 mg TE/g extract with the only exception of the MSY extract which was inactive. Similar results were obtained for the hexane extracts that displayed values ranging from 0.5 to only 6.6 mg CE/g extract, with both LSW and LSM grass pea extracts being totally inactive. Finally, only the dichloromethane extracts of chickpea CAA and grass pea LSW cultivars were determined as actives displaying respectively DPPH^•^ assay values of 2.9 and 2 mg CE/g extracts. The herein presented bioactivities of legumes hay extracts can be rationalized considering the previous results for their bean extracts, which were mostly inactive as a consequence of their low TPC content [26], while the determined herein increased DPPH^•^ activities are directly corelated with their respective elevated TPC values, especially for the methanolic extracts. Nevertheless, among the investigated eight Fabaceae plant extracts as most active was determined the methanolic extract of *V. faba* (cl. Polykarpi/VFP) that displayed a DPPH^•^ value of 84.6 mg TE/g extract. This cultivar also exhibited the highest TPC value.

#### 3.2.2. FRAP Assay

The herein presented results for the reducing capacity of the aerial parts of the investigated eight cultivars present a conformity with the results for their respective beans [26]. Present results also confirmed a similar pattern within extracts, since a low activity was determined for the methanolic extracts, a mild for the hexane extracts and a moderate for the dichloromethane extracts. The latter exhibited FRAP activity values expressed as mg CE/g extract which escalated from 0.64 (CAA) to 0.78 (LSW), 1.66 (PSD), 1.72 (LSM), 1.86 (PSO), 2.03 (PVP), 2,75 (MSY), and 3.41 (VFP). These values are significantly higher compared to those of their respective bean that extracts that ranged from 0.27 (CAA) to 1.77 (VFP) mg CE/g extract [26]. Hexane extracts were less active exhibiting FRAP activity values ranging from 0.28 for CAA to 1.44 for MSY, which are higher than the respective literature results for their bean extracts, which ranged from 0.01 (CAA) to 0.23 (VFP) mg CE/g extract [26]. Finally, the methanolic extracts of the aerial parts were the least active displaying FRAP activity values ranging from 0.07 (MSY) to 0.15 to 1.28 (VFP) mg CE/g extract. In general, it must be noted that the FRAP values determined herein for the aerial parts extracts of investigated legumes exceeded by a magnitude of 7–10 those recorded for their respective bean extracts [26], confirming the good correlation between antioxidant activity and phenolic content. 

With respect to the antioxidant properties of the individual cultivars, as most actives were determined the dichloromethane extracts of *V. faba* (var Polykarpis) and *M. sativa* (cl. Yliki/MSY). This is the first report concerning the antioxidant activity of *V. faba* aerial part, since most of the relevant research has been focused on its seeds, indicating that immature faba seeds display superior antioxidant activity as compared to their respective mature seeds [31] and leaves [32]. On the contrary, the *M. sativa* species comprises the most studied Fabaceae plant for its antioxidant activity [33,34]. 

### 3.3. Fingerprinting of Individual Molecules Presence 

#### 3.3.1. Fatty Acids 

**Figure 4:**
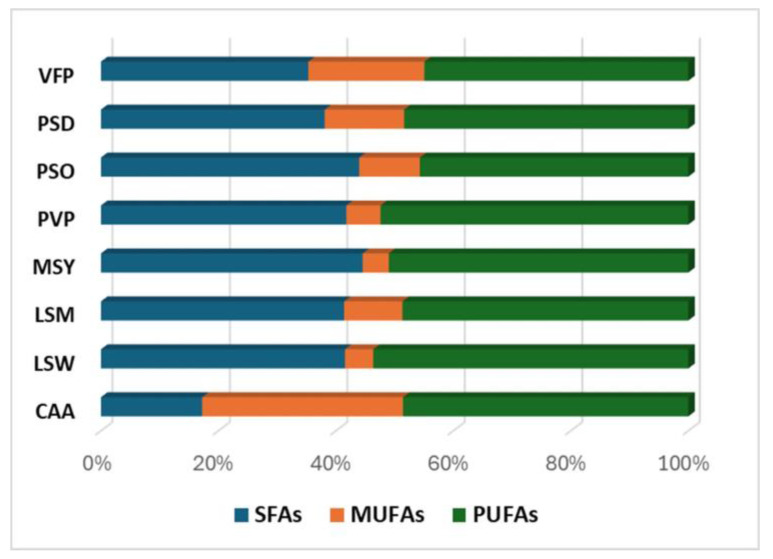
Percentage of saturated (SFA), mono-unsaturated (MUFA) and poly-unsaturated (PUFA)- fatty acids of the Fabaceae plants samples.

In the investigated eight extracts, the presence of 26 different fatty acids was revealed, 16 were classified as saturated fatty acids (SFA), six as mono-unsaturated (MUFA), and four as poly-unsaturated (PUFA). Their cumulative concentration under these three categories is depicted in Figure 4. With respect to the presence of individual molecules, the acids linoleic and linolenic excelled in chickpea (CAA), fava bean (VFP), and in the two grass-pea (LSW, LSM) extracts, with concentrations ranging from 94.7 to 10.7 mg/g extract for linoleic and from 64.1 to 11.8 mg/g extract for linolenic acid. On the other hand, although MUFA compounds were detected in all extracts, they contribute significantly only to chickpea (CAA) and faba bean (VFP) extracts with respective concentrations of 80.2 and 42.9 mg/g extract. Oleic acid is the prevailing MUFA since its presence is ranging from 96.74% (CAA) to 88.17% (PSD), 85.82% (PVP), 84.20% (VFP), 79.44% (PSO), 72.73% (LSW), 66.40% (MSY), and 63.70% (LSM) of their total MUFA content. On the other hand, the abundance and distribution of SFAs are comparable to PUFAs with the notable exception of the chickpea extract (CAA), which was found to contain a significantly lower amount of SFAs. Palmitic acid was the prevailing SFA in all extracts with higher concentrations of 39 mg/g and 38.2 determined for LSW and VFP extracts respectively. To a lesser extent, the acids stearic, arachidic, behenic, and lignoceric acids were also found to contribute also significantly to the SFA profile of the extracts. Stearic and arachidic acid were detected in notable concentrations in the two grass-pea extracts with stearic ranging from 12.9 (LSM) to 16.4 (LSW) mg/g and arachidic from 7.1 (LSM) to 8.4 (LSW) mg/g. The herein defined individual fatty acids profile is in agreement with previous reports determining palmitic, oleic, linoleic, and linolenic fatty acids as most abundant in Fabaceae extracts and confirming their complex chemical diversity [35]. 

The comparison of fatty acids profiles with their respective bean profiles [26] reveals that the investigated hay samples display a similar PUFA placement, followed by MUFA and concluding to smaller concentrations of SFA, with the exclusion of chickpea bean extract, which does not contain PUFAs. The determination of severely reduced amounts of SFAs in beans extracts, as compared to hay samples, is indicative of their potential role in fatty acid circulation. It must be noted that beyond the 23 fatty which have already been detected into beans, herein we have identified the presence of three additional fatty acids in hay extracts. In specific, the SFA undecanoic acid was detected as a minor constituent in the VFP extract, the MUFA *cis*-10-pentadecenoic acid was found in 0.65 and 0.49 mg/g concentrations in MSY and PSO extracts respectively and the PUFA *cis*-11,14,17-eicosatrienoic acid was detected in 0.5 and 0.32 mg/g concentrations into PSD and MSY extracts respectively. 

With respect to the presence of the remaining 23 fatty acids, many previous studies have established the health benefits of the consumption of dietary long-chain *n*-3 fatty acids such as α-linolenic acid. Their intake has been determined as essential for the normal growth and development of living organisms [36]. It is further noticeable that lactating cow’s rations supplementation with high concentrations of palmitic acid change the fatty acid composition of milk lipids modifying their functional properties and resulting in the production of harder butters [37]. 

To date, the fatty acid content of *C. arietinum* aerial parts has not been studied, although an extensive review has summarized efficiently the relevant advances in chickpea beans [38]. On the contrary, the aerial parts of *Lathyrus* sp. have already been studied for their fatty acids’ content by Hanbury et al. [39], which reported the presence of the fatty acids linoleic, linolenic, palmitic, arachidic, oleic, and stearic, in concentrations ranging 14–67%, 1–8%, 8–17%, 0–0.5%, 1–58% and 2–14%, respectively. In 2008, a study concerning the different genotypes of *V. faba*’s leaves and stems indicated that all samples studied were rich in linolenic and linoleic acids [40]. These two acids were also determined as the most abundant in *Medicago sativa* L., var. Giulia [41]. Finally, studies on the fatty acid content of *P. sativum and P. vulgaris* have mainly focused on their seeds and seeds’ oils content [42,43,44,45], highlighting as prevailing the fatty acids palmitic, stearic, oleic, linoleic and *α*-linolenic, with diverse distributions depending on the species and geographical conditions of the investigated seeds [43]. Studies on their beans determined the presence of higher concentrations of the fatty acids *α*-linolenic, linoleic, palmitic, and oleic [42]. Our present findings in the plant tissues are consistently aligned with the respective results for seeds.

#### 3.3.2. Phenolics

Results herein concerning the tracing of 67 phenolic compounds [26,46] detected the presence of 30 phenolics as components of the investigated extracts. Among them, flavonoids comprise the most abundant class of compounds, which are represented by 20 molecules, consisting of 10 isoflavones, six glucoside derivatives and four polyphenols. The respective results are depicted in Table 4. These results though directly related to the TPC results of Table 2, do present some discrepancies presumably because of the limited number of molecules included in the phenolic fingerprinting. The most pronounced discrepancy was traced for the VFP methanolic extract that exceled in TPC content but displayed relatively poor phenolic fingerprint results. This inconsistency suggests that probably several phenolic constituents were not included in the investigated 67 compounds and therefore more research is needed to elucidate this difference. 

The determined herein remarkable structural diversity of Fabaceae herbal extracts mainly concerns only a limited number of plants, since the vast majority of phenolic compounds were detected only in three extracts, namely chickpea (CAA) which contains 21 phenolics and faba (VFP) and kidney (PVP) beans containing respectively 16 and 15 phenolics. The remaining extracts displayed a less diverse and voluminous phenolic content. Finally, it must be underlined that the molecule of isoquercetin was not detected in chickpea extract (CAA), the most diverse polyphenol containing sample, whereas this compound was determined as component in all samples studied. 

With respect to the 19 phenolic compounds identified as components of the respective bean extracts [26], a group of 17 molecules was also detected in the investigated herein hay extracts. Only the molecules of coumestrol and hydroxytyrosol were not detected herein, although these molecules had been identified as components of PVP bean extract. On the other hand, from the phenolics detected in hay extracts the molecules of hesperetin, sissotrin, formononetin, luteolin-4’-O-glucoside, isorhamnetin, and biochanin A were traced only in the chickpea CAA extract. In addition, phenolics detected in only one extract were the molecules of 3’,4’,7-trihydroxyisoflavone (in VFP) and liquiritigenin (PVP), while three additional compounds, namely gallocatechin, quercetin and rutin, were found in both chickpea CAA and kidney bean PVP extracts. Luteolin was determined as component of CAA, PVP and FVP extracts, genistin was found in chickpea CAA and two pea PSO and PSD extracts and diosmin was detected in the grass-pea LSM the pea PSD and the faba bean VFP extracts. The overall comparison of results obtained herein for hay extracts is indicative that these extracts excel in both diversity and volume of contained phenolics in comparison with the related bean extracts. 

With respect to literature reports, it must be noted that until today, the most relevant studies mainly concern the determination of phenolics in legumes seeds. In specific, *C. arietinum* seeds that are known as chickpea and used in human diets are the more extensively studied. Reports on their isoflavone content have revealed the presence of molecules biochanin A, formononetin, ganistein, calycosin, ononin and sissotrin [47]. Study herein constitutes the first report for the phenolic content of *C. arietinum* whole plant and specifically its Greek variety Amorgos. With respect to the remaining hays, the main components of *LSW L. sativus* variety were the molecules of isoquercetin and epigallocatechin gallate and in *L. sativus* (cl. Maleme-107) the molecules of diosmin and chlorogenic acid. 

Previous studies on *M. sativa* species have established the presence of phenolics apigenin, daidzein, diosmetin, chlorogenic acid, caffeic acid, kaempferol, myricetin, and genistin in various concentrations, depending on the different developmental stages of the plant [48]. Herein, we have detected that the aerial part of the investigated *MSY* cultivar contains the first four phenolics, in addition to the presence of molecules isoquercetin, quercitrin and ononin. In *Pisum* samples, the molecule of isoquercetin was the most abundant component. In specific, *PSO* was determined to contain a higher concentration of total phenolics as compared to *PSD*, while *PSD* displayed the richest phenolic diversity. Finally, the aerial part of *P. sativum* plants was determined to contain kaempferol and quercetin glycosides, along with phenolic acids such as caffeic and sinapic acid [49], and flavonoids such as apigenin and luteolin [50].

### 3.4. Hierarchical Clustering Analysis (HCA) and Principal Component Analysis (PCA)

Hierarchical clustering analysis (HCA) is a powerful statistical method frequently used to elucidate patterns in the relationships between various genetic materials based on their phytochemical traits. Combining fatty acid composition, polyphenol content, and antioxidant assays resulted in a classification that demonstrated stronger grouping between cultivars of the same species compared to what was previously observed in the relevant data for their respective bean extracts [26].

Principal component analysis (PCA) facilitates the simultaneous understanding of distinguishing traits that explain genetic variability and their interrelationships. In this study, both PCAs produced a stronger grouping by genera than in the previous study.

These findings reveal the potential utilities arise from the identification of the phytochemical profiles of the byproducts, with respect to the value of the respective data and information for breeders in the selection of the best genotypes. The phytochemical traits and antioxidant assays in both studies explained a significant percentage of the variability, indicating that selecting individuals with traits that have high score loadings on the first principal components can effectively exploit genetic variability.

Finally, the highlight of VFP extract by both approaches as a distinctive case can be rationalized considering that all VFP extracts displayed increased bioactivity which was utilized as an essential correlation parameter. 

## 4. Materials and Methods

### 4.1. Samples

The study is focused on six Fabaceae *taxa*, represented by eight novel Greek cultivars provided by the Institute of Industrial and Forage Crops (IIFC) of the Hellenic Agricultural Organization ELGO-DIMITRA. The plants were grown in replicated field trials at the central farm of IIFC in Larissa (39°36′ N, 22°25′ E) during the cultivation period of 2020–2021. At the suitable stage of optimum hay production of each species (pod setting stage), plants from each plot were cut to ground level with manual shears and their fresh weight was recorded. A sample of 2.0 kg from each plot was dried at 70°C for 72 h for further analyses. The samples investigated belong to the following species: *Cicer arietinum* L. (cv. Amorgos), *Lathyrus sativus* L. (Inbred line LSW and cl. Maleme-107), *Medicago sativa* L. (cl. Yliki), *Phaseolus vulgaris* L. (cl. Pirgetos), *Pisum sativum* L. (cls. Olympos and Dodoni), and *Vicia faba* L. (cl. Polykarpi).

### 4.2. Chemicals and Standards 

Analytical grade solvents hexane, dichloromethane and methanol were obtained from Fisher Chemicals (Hampton, NH, USA) and were used as extraction solvents. The LC–MS/MS grade quality solvents water and acetonitrile were purchased from J.T. Baker (Phillipsburg, NJ, USA), while the LC–MS grade formic acid was obtained from Fisher Chemicals. Potassium hydroxide (KOH) and 14% in methanol boron trifluoride (BF_3_) were purchased from Sigma-Aldrich (Burlington, MA, USA) and hydrochloric acid (HCl) was obtained from Fluka (Long Island, NY, USA).

In total 67 phenolic standards were used for the LC-MS/MS determination. They were purchased from Extra-Synthese (Genay, France) except puerarin and equol which were obtained from TCI (Tokyo Chemical Industry) (Tokyo, Japan) and calycosin, calycosin-7-O-β-D-glucoside, lariciresinol, matairesinol, and secoisolariciresinol which were provided from Biosynth Carbosynth (Gardner, MA, USA). All standards’ purity was >95%, expect 3′,4′,7-trihydroxyflavone (purity >90%). For the LC-MS/MS polyphenols’ determination, 2-(4-chlorophenyl) malonaldehyde was used as internal standard, which was provided by Sigma-Aldrich. The Supelco 37 Component FAME Mix obtained from Sigma-Aldrich (St. Louis, MO, USA) was used for the detection and quantification of the fatty acids presence. 

### 4.3. Samples Preparation 

Immediately after their collection, plant biomass samples were placed in a dark and well-ventilated place to dry. The dried samples were powdered and extracted successively with n-hexane, dichloromethane and methanol as previously described by Myrtsi et al. [15]. Solvent removal was performed using a Büchi vacuum pump V-700, Vacuum controller V-850 (Büchi, Flawil, Switzerland) rotary evaporator equipped with a Julabo F12 cooling unit (Julabo, Seelbach, Germany), in temperatures below 35°C. The determination of fatty acids and polyphenols was performed on hexane and methanolic extracts respectively [10].

### 4.4. Determination of Total Phenolic (TPC) & Tannin (TTC) Content 

The detailed spectrophotometric methods applied for TPC and TTC determinations have been reported previously [24]. Briefly, all samples were placed in a 96-well microplate (Sarstedt AG & Co. KG, Nümbrecht, Germany) in triplicate and the respective absorbance was measured in a NanoQuant, infinite M200PRO (Tecan Group Ltd., Männedorf, Switzerland) instrument. The TPC was determined at 765 nm absorption wavelength and the respective value was obtained against a gallic acid standard calibration curve. The respective results were expressed as mg of gallic acid equivalents per g of extract (mg GAE/g extract). The TTC was measured at 500 nm absorption wavelength and its value was calculated using a catechin standard calibration curve. The respective results are expressed as mg of catechin equivalents per g of extract (mg CE/g extract) [15].

### 4.5. Determination of Individual Phenols Content by LC–MS/MS Analysis 

The individual phenols content was determined using an Accela Ultra High-Performance Liquid Chromatography system coupled with a TSQ Quantum Access triple quadrupole mass spectrometer (Thermo Fisher Scientific, Inc., Waltham, MA, USA), as previously described by Myrtsi et al. [46]. In particular, the analytes’ chromatographic separation was achieved on a C18 column (150 × 2.1 mm, 3 μm, Fortis Technologies Ltd., Neston, Cheshire, UK), which was coupled with an AF C18 guard column (10 × 2.0 mm, 3 μm, Fortis Technologies Ltd., Neston, Cheshire, UK) using a mobile phase consisted of A: water with 0.1% formic acid and B: 100% acetonitrile. The ElectroSpray Ionization (ESI) technique was used for the MS/MS determination and the determinations were performed at Selected Reaction Monitoring mode (SRM). The quantification of polyphenols was achieved by constructing a calibration curve for each analyte using six concentration levels for each standard solution, along with an internal standard solution. The respective parameters of transition, collision energy, polarity, retention time, calibration curve equation, and determination coefficient for each analyte have been recently reported [26]. Finally, the respective LOD and LOQ values for each analyte are provided in the Supplementary Materials (Table S2).

### 4.6. Determination of Fatty Acids Content by GC-FID Analysis

The detection and quantitation of fatty acids was performed on hexane extracts using a 7820A GC-FID System (Agilent Technologies, Inc., Santa Clara, CA, USA) instrument equipped with a DB-WAX 30 m, 0.25 mm, 0.25 µm (Agilent Technologies, Inc., Santa Clara, CA, USA) column. Before the analysis, it was necessary for the fatty acids were esterified. The detailed parameters (retention time, calibration curve equation, and determination coefficient of each fatty acid) have been reported previously by Myrtsi et al. [15,26] and provided as Table S3 in the Supplementary Materials.

### 4.7. Antioxidant Properties Evaluation

The FRAP (Ferric Reducing Antioxidant Power) and DPPH^•^ (Radical Scavenging Assay assays were used for the determination of samples’ antioxidant properties. The respective assessments were performed using a NanoQuant, infinite M200PRO (Tecan Group Ltd., Männedorf, Switzerland) instrument and following previously reported procedures [15,26]. For the FRAP assay, the absorbance was measured at 593 nm wavelength and the reducing capacity was determined against a FeSO_4_ standard calibration curve. The respective results have been expressed as mmol Fe^2+^/g of extract. For the DPPH^•^ assay, the absorbance was measured at 515 nm wavelength and the antioxidant activity was determined against a Trolox calibration standard curve. The respective results were expressed as mg Trolox Equivalents (TE)/g of extract [10].

### 4.8. Statistical Analysis

The statistical functions of Microsoft Office 365 were used. All results are presented as mean value ± standard deviation (SD) of experiments that were performed in triplicate. For all calculations performed in this work, the Durbin–Watson (DW) statistical tests for the residuals, the οne-way analysis of variance (ANOVA), and the *t*-test were used. The calculated *p*-value was always less than 0.05.

Using the full linkage method, hierarchical cluster analysis was utilized to calculate the Euclidean distances of legume hays according to their antioxidant and phytochemical properties. In addition, the original variables’ dimensionality was decreased by using principal component analysis (PCA). The statistical analyses were conducted using R version 4.3.2 (www.R-project.org, accessed on 20 May 2024) and the R packages, dendetxend [51] and Factominer [52]. 

## 5. Conclusions

Hay samples obtained from eight novel Greek cultivars belonging to Fabaceae family plants were thoroughly investigated with respect to their phytochemical content and bioactivity. The phytochemical investigation concerned the assessment of their phenolic, tannin and fatty acids content, while the bioactivity assessment referred to the determination of their antioxidant capacity. 

Most samples studied were determined to contain a significant diversity of phytochemicals, further specified as the fatty acids content of their hexane extracts and the phenolics found into their methanolic extracts. On the other hand, their content of antinutrient tannins was low while all extracts exhibited potent antioxidant properties. 

Among the species and cultivars tested herein, *V. faba*/cl. Polykarpi displayed the highest total phenolic content, while *C. arietinum*/cl. Amorgos exhibited the highest diversity of studied polyphenols. These results were verified by the two antioxidant capacity tests which indicated that *V. faba* display the most significant antioxidant potency. On the contrary, six of the investigated samples did not find to contain tannins, which are known antinutrients. Finally, the presence of 26 fatty acids was revealed in plant samples, composed of 16 saturated, six mono-unsaturated and four poly-unsaturated fatty acids. 

The determined phytochemical content in combination with the increased antioxidant capacities and the deprived amount of anti-nutrient tannins are indicative of their potency for the incorporation of hays of these novel cultivars in livestock feeding. Furthermore, they advocate the inclusion of the proposed investigation in the development of new cultivars since the detected differences between novel-improved cultivars and their respective traditional cultivars are indicative of their advantages and the significance of breeding in plant biochemistry. 

## Figures and Tables

**Table 1:** Legume cultivars aerial parts extraction yield (%).

Code	Sample	Extraction Yield (%)		
		Hex	DCM	MeOH
CAA	*Cicer arietinum* (cv. Amorgos)	1.61	0.95	9.79
LSW	*Lathyrus sativus* (Inbred line)	1.65	1.29	21.06
LSM	*Lathyrus sativus* (cv. Maleme-107)	1.06	1.12	17.58
MSY	*Medicago sativa* (var Yliki)	2.24	1.94	15
PVP	*Phaseolus vulgaris* (cv. Pirgetos)	1.09	1.25	10.84
PSO	*Pisum sativum* (cv. Olympos)	6.8	1.53	12.98
PSD	*Pisum sativum* (cv. Dodoni)	2.05	1.27	10.48
VFP	*Vicia faba* (cv. Polykarpi)	0.85	0.87	12.35

**Table 2:** Total Phenolic (TPC) and Tannin (TTC) content of the investigated Fabaceae biomass methanolic extracts.

Samples	TPC (mg GAE/g Extract)	TTC (mg CE/g Extract)
CAA	14.2 ± 0.2 **	<LOD
LSW	13.1 ± 0.1 *	<LOD
LSM	11.1 ± 0.1 **	<LOD
MSY	11.4 ± 0.4 *	<LOD
PVP	18.9 ± 1.4 *	<LOD
PSO	19.5 ± 1.2 *	25.3 ± 14.6 *
PSD	16.7 ± 0.8 *	<LOD
VFP	46.9 ± 0.2 **	7.7 ± 4.5 *

GAE: gallic acid equivalent; CE: catechin; <LOD: below the limit of detection; *p*-value correlating samples with the corresponding standard solution: * *p* ≤ 0.005, ** *p* ≤ 0.01.

**Table 3:** Outcome of the DPPH^•^ and FRAP antioxidant assays for the investigated Fabaceae extracts.

Samples	DPPH (mg TE/g Extract)			FRAP (mmol Fe(II)-E/g Extract)		
	Hex	DCM	MeOH	Hex	DCM	MeOH
CAA	0.5 ± 0.3 **	2.9 ± 1.4 **	0.4 ± 0.1 *	0.28 ± 0.00 *	0.64 ± 0.02 *	0.18 ± 0.00 *
LSW	<LOD	2.0 ± 0.9 *	3.2 ± 0.5 *	0.58 ± 0.01 *	0.78 ± 0.02 *	0.15 ± 0.00 *
LSM	<LOD	<LOD	2.1 ± 0.7 *	0.65 ± 0.01 *	1.72 ± 0.03 *	0.18 ± 0.00 *
MSY	1.5 ± 0.6 *	<LOD	<LOD	1.44 ± 0.04 *	2.75 ± 0.04 *	0.07 ± 0.00 *
PVP	1.1 ± 0.8 *	<LOD	12.5 ± 1.1 *	1.12 ± 0.05 *	2.03 ± 0.03 *	0.28 ± 0.01 *
PSO	6.6 ± 0.0 *	<LOD	17.4 ± 9.9 *	0.32 ± 0.02 *	1.86 ± 0.03 *	0.33 ± 0.00 *
PSD	1.4 ± 0.8 *	<LOD	6.2 ± 0.2 *	0.89 ± 0.03 *	1.66 ± 0.02 *	0.19 ± 0.00 *
VFP	5.6 ± 0.7 *	<LOD	84.6 ± 8.3 *	0.62 ± 0.02 *	3.41 ± 0.07 *	1.28 ± 0.00 *

TE: Trolox equivalent; Fe(II)-E: Fe(II) equivalent; <LOD: below the limit of detection; *p*-value correlating samples with the corresponding standard solution: * *p* ≤ 0.005, ** *p* ≤ 0.01.

**Table 4:** Phenols contained in the exploited Fabaceae plants (mg/g methanolic extract).

Polyphenol	CAA	LSW	LSM	MSY	PVP	PSO	PSD	VFP
3’,4’,7-Trihydroxyisoflavone	-	-	-	-	-	-	-	tr
Luteolin-4’-O-glucoside	0.113 ± 0.004 *	-	-	-	-	-	-	-
Quercetagetin-7-O-glucoside	-	-	tr	-	tr	tr	-	tr
Apigenin	0.004 ± 0.000 ***	-	-	0.154 ± 0.000 ***	tr	-	-	0.009 ± 0.000 ****
Biochanin A	0.150 ± 0.006 ***	-	-	-	-	-	-	-
Epigallocatechin gallate	-	tr	tr	-	-	-	-	tr
Gallocatechin	0.004 ± 0.001 *	-	-	-	0.010 ± 0.001 *	-	-	-
Genistein	0.017 ± 0.001 ***	-	-	-	0.055 ± 0.001 **	-	-	0.383 ± 0.005 ***
Glycitein	0.013 ± 0.005 *	-	-	-	tr	-	0.001 ± 0.000 *	tr
Genistin	0.013 ± 0.001 **	-	-	-	-	tr	tr	-
Daidzin	tr	-	-	-	tr	-	-	-
Daidzein	0.001 ± 0.000 *	-	-	tr	0.033 ± 0.000 *	tr	-	-
Diosmetin	0.229 ± 0.002 *	-	-	0.006 ± 0.001 *	tr	-	-	0.006 ± 0.000 *
Diosmin	-	-	0.051 ± 0.003 *	-	-	-	0.004 ± 0.001 **	0.013 ± 0.001 *
Hesperetin	tr	-	-	-	-	-	-	-
Isoquercetin	0.365 ± 0.023 *	tr	-	0.003 ± 0.001 *	0.020 ± 0.001 *	0.033 ± 0.003 *	0.012 ± 0.002 *	0.001 ± 0.000 *
Isoliquiritigenin	0.004 ± 0.001 *	-	-	-	0.002 ± 0.000 *	0.002 ± 0.000 *	0.002 ± 0.000 *	0.008 ± 0.000 *
Isorhamnetin	tr	-	-	-	-	-	-	-
Calycosin	0.062 ± 0.012 ****	-	tr	-	-	0.002 ± 0.000 *	0.004 ± 0.000 *	tr
Quercetin	0.226 ± 0.003 *	-	-	-	0.121 ± 0.004 *	-	-	-
Quercitrin	-	-	tr	tr	0.001 ± 0.000 *	-	-	tr
Liquiritigenin	-	-	-	-	tr	-	-	-
Luteolin	0.017 ± 0.002 *	-	-	-	0.025 ± 0.001 *	-	-	0.012 ± 0.000 *
Ononin	0.041 ± 0.011 **	-	-	tr	-	-	tr	-
Procyanidin B1	-	-	-	-	-	-	-	0.001 ± 0.000 *
Procyanidin B2	-	-	-	-	-	-	-	tr
Rutin	0.009 ± 0.003 *	-	-	-	0.090 ± 0.006 *	-	-	-
Sissotrin	0.165 ± 0.048 *	-	-	-	-	-	-	-
Formononetin	0.092 ± 0.001 *	-	-	-	-	-	-	-
Chlorogenic acid	-	-	0.005 ± 0.002 *	tr	-	tr	0.003 ± 0.002 *	0.011 ± 0.001 *

tr: <0.001 mg/g; nd: not detected; *p*-value correlating samples with corresponding standard solution: * *p* ≤ 0.005, ** *p* ≤ 0.01, *** *p* ≤ 0.03, **** *p* ≤ 0.05.

## Data Availability

The data are contained within the article.

## Supplementary Materials

The following supporting information can be downloaded at: https://www.mdpi.com/article/10.3390/plants13213058/s1, Table S1: Fatty acids composition in Fabaceae plants’ samples (mg/g extract); Table S2: Transition, collision energy, polarity, retention time (RT), calibration curve equation and determination coefficient, LOD, and LOQ of each analyte; Table S3: Retention time (RT), calibration curve equation and determination coefficient (R^2^) of each fatty acid. Figure S1: Correlation heatmap depicting the relationships between phytochemical components and antioxidant properties.
