# Supplementary material for: Hays of Novel-Improved Legume Cultivars: Phytochemical Content and Nutritional Value
Source: Plants (Basel). 2024 Oct 31;13(21):3058. doi: 10.3390/plants13213058 (PMC11548321; doi:10.3390/plants13213058)
Supplement: Supplementary file 1 [file plants-13-03058-s001.zip › plants-3127934-supplementary.pdf]

Hays of Novel-Improved Legume Cultivars: Phytochemical Content and Nutritional Value

Eleni D. Myrtsi, Dimitrios N. Vlachostergios, Christos Petsoulas, Epameinondas Evergetis, Sofia D. Koulocheri and Serkos A. Haroutounian*

Supplementary Material

Table S1. Fatty acids composition in *Fabaceae* plants' samples (mg/g extract).

Table S2. Transition, collision energy, polarity, retention time (RT), calibration curve equation and determination coefficient, LOD, and LOQ of each analyte.

Table S3. Retention time (RT), calibration curve equation and determination coefficient (R^2) of each fatty acid.

Figure S1. Correlation heatmap depicting the relationships between phytochemical components and antioxidant properties.

Table S1. Fatty acids composition in *Fabaceae* plants' samples (mg/g extract).

**Fatty Acids**	**CAA**	**LSW**	**LSM**	**MSY**	**PVP**	**PSO**	**PSD**	**VFP**
Caproic acid	0.179±0.003	0.316±0.022	0.266±0.007	0.318±0.018	0.512±0.034	0.091±0.001	0.079±0.007	0.584±0.025
Caprylic acid	0.085±0.008	0.128±0.006	0.274±0.006	0.138±0.010	0.217±0.012	-	0.049±0.002	0.700±0.040
Capric acid	0.086±0.02	0.028±0.009	0.178±0.006	0.145±0.012	0.212±0.011	0.003±0.000	0.047±0.006	0.520±0.032
Undecanoic acid	-	-	-	-	-	-	-	0.041±0.003
Lauric acid	0.674±0.032	0.510±0.041	0.551±0.011	2.097±0.141	0.837±0.010	0.332±0.009	0.396±0.043	2.064±0.121
Tridecanoic acid	-	-	0.024±0.002	0.024±0.004	0.006±0.004	-	0.003±0.003	0.167±0.012
Myristic acid	1.711±0.089	2.102±0.167	2.578±0.042	3.123±0.278	2.729±0.171	0.926±0.016	1.302±0.150	3.283±0.163
Pentadecanoic acid	0.193±0.014	0.583±0.056	0.769±0.010	0.547±0.045	0.427±0.042	0.280±0.007	0.449±0.062	0.749±0.025
cis-10-Pentadecenoic acid	-	-	-	0.653±0.029	-	0.494±0.015	-	-
Palmitic acid	28.139±1.371	39.009±3.025	34.715±0.371	33.398±2.459	31.294±4.211	10.578±0.127	15.063±2.025	38.319±2.074
Palmitoleic acid	0.600±0.033	0.201±0.029	0.490±0.001	0.274±0.022	0.307±0.046	0.101±0.001	0.385±0.078	0.393±0.018
Margaric acid	0.199±0.007	1.008±0.087	1.427±0.189	0.751±0.053	0.432±0.087	0.328±0.006	0.511±0.070	1.277±0.05
cis-10-Heptadecenoic acid	0.128±0.008	-	-	0.187±0.106	-	-	-	0.141±0.008
Stearic acid	4.763±0.03	16.364±1.296	12.898±0.117	7.374±0.558	6.84±1.424	4.395±0.049	5.871±0.773	11.041±0.311
Oleic acid	77.578±2.185	6.377±0.522	10.423±0.093	3.841±0.288	6.252±1.254	4.042±0.008	9.356±1.276	36.112±1.218
Linoleic acid	94.717±2.622	32.743±2.553	44.598±1.082	24.984±1.83	26.722±5.384	10.741±0.124	22.799±2.925	68.451±2.388
Linolenic acid	19.049±0.454	64.132±4.839	35.35±0.345	40.890±2.932	38.860±7.706	11.752±0.124	14.496±1.929	28.972±1.125
Arachidic acid	1.914±0.043	8.362±0.635	7.112±0.004	3.729±0.119	3.241±0.753	2.027±0.002	2.437±0.291	7.320±0.220
cis-11-Eicosenoic acid	1.680±0.047	0.264±0.027	3.187±0.132	-	-	-	0.283±0.027	4.357±0.038
cis-11,14-Eicosadienoic acid	0.137±0.002	0.432±0.156	0.267±0.001	0.085±0.017	-	-	0.170±0.019	0.307±0.013
Heneicosanoic acid	0.387±0.001	1.275±0.088	1.366±0.004	0.524±0.020	0.458±0.075	0.683±0.005	0.684±0.075	1.656±0.044
cis-11,14,17-Eicosatrienoic acid	-	-	-	0.316±0.062	-	-	0.503±0.043	-
Behenic acid	0.269±0.014	2.617±0.205	2.421±0.012	2.542±0.164	1.640±0.387	0.926±0.037	1.456±0.204	3.143±0.072
Erucic acid	0.206±0.007	1.662±0.044	2.262±0.013	0.830±0.054	0.726±0.121	0.451±0.031	0.587±0.066	1.886±0.087
Tricosanoic acid	0.552±0.010	1.021±0.06	1.614±0.317	0.728±0.042	0.803±0.173	0.474±0.023	0.632±0.09	1.684±0.079
Lignoceric acid	1.256±0.026	2.056±0.255	2.068±0.545	2.484±0.12	2.649±0.511	0.591±0.049	0.917±0.147	4.257±0.031
SFAs (%** of total Fatty Acids**)	17.23	41.54	41.41	44.56	41.78	44	38.1	35.32
MUFAs (%** of total Fatty Acids**)	34.2	4.832	9.926	4.451	5.82	10.3	13.5	19.73
PUFAs ((%** of total Fatty Acids**)	48.57	53.63	48.66	50.99	52.4	45.7	48.4	44.95

Table S2. Transition, collision energy, polarity, retention time (RT), calibration curve equation, determination coefficient, LOD, and LOQ of each analyte.

Compound	Parent Mass	Product Mass	Collision Energy (eV)	Polarity	RT (min)	Equation	R^2	LOD (ng/mL)	LOQ (ng/mL)
Phenolic acids									
Caffeic acid	180.046	135.902	19	(−)	3.89	$y=2.45545\times10^{-05}+0.807048x$	0.9997	51.4	155.8
Chlorogenic acid	354.158	191.689	22	(−)	2.36	$y=0.00237338+0.0463491x$	0.9992	44.6	135.1
Gallic acid	170.064	126.042	18	(−)	2.05	$y=-0.000909657+0.248304x$	0.9996	73.1	221.5
Neochlorogenic acid	354.180	190.875	21	(−)	2.04	$y=0.00110944+0.330727x$	0.9996	12.6	38.2
p-Coumaric acid	163.994	119.995	18	(−)	6.16	$y=0.00253174+0.386656x$	0.9998	46.0	139.5
Protocatechuic acid	154.055	109.992	18	(−)	2.37	$y=0.00114193+0.336255x$	0.9996	16.2	49.1
Sinapic acid	224.081	208.913	16	(−)	6.78	$y=0.00292367+0.00171603x$	0.9999	54.9	166.2
Flavonoids									
Apigenin	269.982	118.164	42	(−)	16.98	$y=-0.000697875+1.44417x$	0.9995	8.7	26.4
Catechin	290.147	204.072	21	(−)	2.9	$y=0.0467805+0.934157x$	0.9996	75.5	228.9
Diosmetin	300.276	284.940	20	(−)	17.67	$y=-0.0358838+5.72414x$	0.9993	53.9	163.3
Diosmin	608.363	607.571<br>284.091	13	(−)	8.1	$y=-0.000612353+0.15086x$	0.9998	12.5	37.7
Epicatechin	290.159	246.261	18	(−)	3.57	$y=-0.116752+4.45105x$	0.9995	18.9	57.3
Epigallocatechin	306.193	125.032	29	(−)	2.47	$y=0.000673414+0.0284518x$	0.9997	39.2	118.9
Epigallocatechin gallate	458.179	168.798	21	(−)	3.44	$y=-0.0193777+0.909754x$	0.9993	30.7	93.1
Eriodictyol	288.199	151.221	17	(−)	12.67	$y=-0.00580681+0.335251x$	0.9996	23.6	71.6
Gallocatechin	306.149	125.017	24	(−)	2.23	$y=6.84895\times10^{-6}+0.223507x$	0.9997	109.5	331.8
Hesperidin	610.143	301.669	26	(−)	7.78	$y=-0.000990985+0.200653x$	0.9999	27.2	82.3
Hesperetin	302.109	286.972	20	(−)	17.37	$y=-0.00283134+2.37119x$	1.0000	13.8	42.0
Isoquercetin	464.047	300.702	27	(−)	6.42	$y=-9.13155\times10^{-5}+0.017002x$	0.9996	46.2	140.0
Isorhamnetin	316.080	300.976	24	(−)	18.08	$y=3.31994\times10^{-5}+0.0163076x$	0.9991	47.5	144.0
Kaempferol	286.099	240.018	31	(−)	17.46	$y=0.00954223+0.0127002x$	0.9999	15.0	45.5
Liquiritigenin	256.166	135.040	18	(−)	12.64	$y=0.0106373+0.516127x$	0.9997	77.4	234.7

Compound	Parent Mass	Product Mass	Collision Energy (eV)	Polarity	RT (min)	Equation	R^2	LOD (ng/mL)	LOQ (ng/mL)
Liquiritin	418.213	256.024	24	(−)	5.96	y=−0.000116163+0.787945x	0.9994	26.3	79.6
Luteolin	286.139	132.980	36	(−)	13.74	y=0.000771555+1.10749x	0.9991	20.6	62.3
Luteolin-4'-O-glucoside	448.270	284.878	21	(−)	8.62	y=0.220464+28.9004x	0.9994	29.8	90.3
Myricetin	318.121	150.835	26	(−)	9.85	y=−0.00277692+0.21372x	0.9992	67.1	203.2
Pelargonidin	271.042	121.024	33	(+)	5.20	y=−0.1191133+10.7511356x	0.9995	23.3	70.5
Pelargonin	595.241	270.881	33	(+)	2.05	y=0.709165842+229.621287x	0.9998	7.1	21.4
Procyanidin B1	578.342	407.739	25	(−)	2.3	y=–0.00010714+0.0256914x	0.9995	46.9	142.2
Procyanidin B2	578.319	408.253	29	(−)	2.83	$y=-5.53133\times10^{-5}+0.03031x$	0.9995	55.0	166.8
Quercetagetin	318.121	139.101	32	(−)	8.49	y=−0.0184825+1.55459x	0.9993	61.1	185.2
Quercetagetin-7-O-glucoside	480.253	317.676	25	(−)	3.91	y=−0.0195021+1.5577x	0.9996	29.8	90.2
Quercetin	302.104	151.049	25	(−)	13.74	y=0.000893219+0.280044x	0.9995	17.9	54.3
Quercitrin	448.181	300.721	28	(−)	8.3	y=−0.00056989+0.0201204x	0.9995	38.5	116.8
Rhamnetin	316.116	165.526	25	(−)	20.58	$y=-2.61552\times10^{-5}+0,0153129x$	0.9990	13.3	40.3
Rutin	610.299	300.561	36	(−)	5.38	y=0.0749463+3.00856x	0.9996	102.2	309.5
Taxifolin	304.097	285.981	15	(−)	7.19	y=−0.000156342+0.241233x	0.9998	17.2	52.2
Isoflavonoids									
3'.4'.7-trihydroxyisoflavone	270.133	242.060	24	(−)	9.01	y=−0.00283483+0.618479x	0.9991	21.8	65.9
4'.6.7-Trihydroxyisoflavone	270.124	240.996	31	(−)	9.66	y=−0.00213213+0.379464x	0.9984	1.8	5.5
Biochanin A	284.126	268.756	21	(−)	25.08	y=0.000605632+3.02904x	0.9995	11.0	33.2
Calycosin	284.134	268.921	21	(−)	13.29	y=−0.0561721+5.79216x	0.9998	22.2	67.3
Calycosin-7-O-D-glycoside	446.305	445.555<br>368.002	14	(+)	10.97	y=−0.000130129+0.0799105x	0.9996	21.8	66.2
Daidzein	254.287	253.568	15	(−)	12.12	y=−0.000638938+2.11645x	0.9995	51.3	155.3
Daidzein-7-O-glucuronide	430.263	253.987	32	(−)	4.15	y=0.000207851+0.125476x	0.9991	52.0	157.4
Daidzin	416.715	254.790	26	(+)	4.02	y=0.000401047+0.0236351x	0.9991	57.1	173.0
Equol	242.265	121.522	17	(−)	16.38	y=−0.00044859+0.00040563x	0.9991	1681.4	5095.2
Formononetin	268.159	252.919	23	(−)	19.69	y=0.00594119+2.83806x	0.9992	30.5	92.4
Genistein	270.132	133.827	37	(−)	16.66	y=0.0236043+0.993835x	0.9991	18.1	54.8

Compound	Parent Mass	Product Mass	Collision Energy (eV)	Polarity	RT (min)	Equation	R^2	LOD (ng/mL)	LOQ (ng/mL)
Genistein-7-O-glucuronide	446.250	268.842	31	(−)	7.32	y=−0.0350294+0.0875658x	0.9999	47.1	142.7
Genistin	432.258	268.887	32	(−)	8.43	y=−0.000522399+0.187494x	0.9992	12.4	37.6
Glycitein	284.179	268.649	24	(−)	13.42	y=−0.000280969+2.77789x	0.9994	10.7	32.5
Glycitin	446.797	284.768	26	(+)	5.21	y=−4.05352+1140.78x	0.9991	70.5	213.7
Ononin	430.731	268.785	21	(+)	10.24	y=—0.00378502+0.19401x	0.9999	20.8	62.9
Puerarin	416.220	295.606	24	(−)	2.79	y=−0.0588534+3.08771x	0.9998	47.5	144.0
Sissotrin	446.327	283.857	22	(−)	14.06	y=−0.00038752+0.0815812x	0.9996	33.8	102.3
Sophoricoside	432.293	431.568<br>270.134	19	(−)	8.3	y=0.002776293+1.036527x	0.9997	20.5	62.1
Chalconoids									
Isoliquiritigenin	256.154	120.019	29	(−)	19.67	y=−0.000802473+1.59052x	0.9996	13.7	41.4
Phloretin	274.125	167.912	19	(−)	16.27	y=−0.000383945+1.55783x	0.9997	20.6	62.3
Phloridzin	436.243	273.890	19	(−)	9.13	y=−0.011024+1.32396x	0.9991	32.4	98.2
Xanthoxumol	354.240	234.027	21	(−)	31.82	y=−0.00111669+0.00335894x	0.9999	81.4	246.6
Lignans									
Lariciresinol	360.220	329.991	12	(−)	9.98	y=0.0036846+0.00281203x	0.9999	42.6	129.1
Matairesinol	358.248	342.987<br>82.914	23	(−)	15.88	y=0.00292574+0.0239047x	0.9992	72.2	218.9
Secoisolariciresinol	362.276	361.511<br>165.755	11	(−)	9.4	y=0.00211407+0.247818x	0.9988	98.8	299.4
Coumestan									
Coumestrol	268.134	239.903	26	(−)	17.23	y=0.000117777+0.39815x	0.9995	4.0	12.1
Phenylethanoil									
Hydroxytyrosol	154.132	124.083	17	(−)	2.25	y=0.000895668+0.106669x	0.9992	33.8	102.3
Stilbenoids									
Polydatin	390.231	227.693	20	(−)	5.94	y=−0.0120849+4.16746x	0.9991	39.4	119.4
Reveratrol	228.014	184.140	24	(−)	11.41	y=0.00125676+0.00646586x	0.9992	120.5	365.1
Internal Stantard	182.748	136.996	26	(−)	15.68				

Table S3. Retention time (RT), calibration curve equation, and determination coefficient (R^2) of each fatty acid.

Fatty Acid	RT (min)	Equation	R^2
Butyric Acid	4.10	y=5.43776+1.81190x	0.9990
Caproic acid	5.21	y=−0.32585+2.19335x	0.9990
Caprylic acid	6.33	y=1.83230+2.45992x	0.9992
Capric acid	7.40	y=4.62271+2.62696x	0.9990
Undecanoic acid	7.95	y=2.18465+2.72111x	0.9990
Lauric acid	8.56	y=3.92115+2.76115x	0.9994
Tridecanoic acid	9.23	y=3.78551+2.70616x	0.9992
Myristic acid	10.04	y=11.43893+2.62566x	0.9991
Myristoleic acid	10.52	y=5.60554+2.62292x	0.9991
Pentadecanoic acid	10.95	y=6.88948+2.58981x	0.9990
cis-10-Pentadecenoic acid	11.64	y=6.53148+2.60193x	0.9990
Palmitic acid	11.98	y=17.49106+2.59396x	0.9992
Palmitoleic acid	12.26	y=6.01822+2.57637x	0.9992
Margaric acid	12.97	y=5.61680+2.52989x	0.9990
cis-10-Heptadecenoic acid	13.26	y=5.60661+2.58709x	0.9990
Stearic acid	14.10	y=11.61671+2.58183x	0.9993
Oleic acid	14.39	y=16.92763+2.57653x	0.9992
Linoleic acid	15.05	y=6.21919+2.50695x	0.9993
Linolenic acid	15.49	y=3.36202+2.43641x	0.9994
Linolelaidic acid	16.01	y=3.05237+2.44261x	0.9993
Arachidic acid	17.01	y=5.38288+2.61615x	0.9994
cis-11-Eicosenoic acid	17.44	y=3.58500+2.57952x	0.9995
cis-11,14-Eicosadienoic acid	18.34	y=1.96961+2.50940x	0.9990
Heneicosanoic acid	18.84	y=−8.40315+1.84368x	0.9991
cis-11,14,17-Eicosatrienoic acid	19.28	y=−11.10622+1.42617x	0.9983
cis-5,8,11,14,17-Eicosapentaenoic acid	19.59	y=4.48371+2.38905x	0.9991
Arachidonic acid	20.75	y=4.24675+2.28699x	0.9991
Behenic acid	20.77	y=8.15731+3.70587x	0.9990
Erucic acid	21.27	y=4.69676+2.52971x	0.9991
cis-13,16-Docosadienoic aci	22.37	y=6.27324+2.41700x	0.9994
Tricosanoic acid	23.09	y=1.88253+2.54624x	0.9997
Lignoceric acid	25.99	y=3.94197+2.51159x	0.9991
cis-4,7,10,13,16,19-Docosahexaenoic acid	26.77	y=−8.77566+3.26195x	0.9982
Nevronic Acid	26.88	y=−9.56931+1.30621x	0.9982

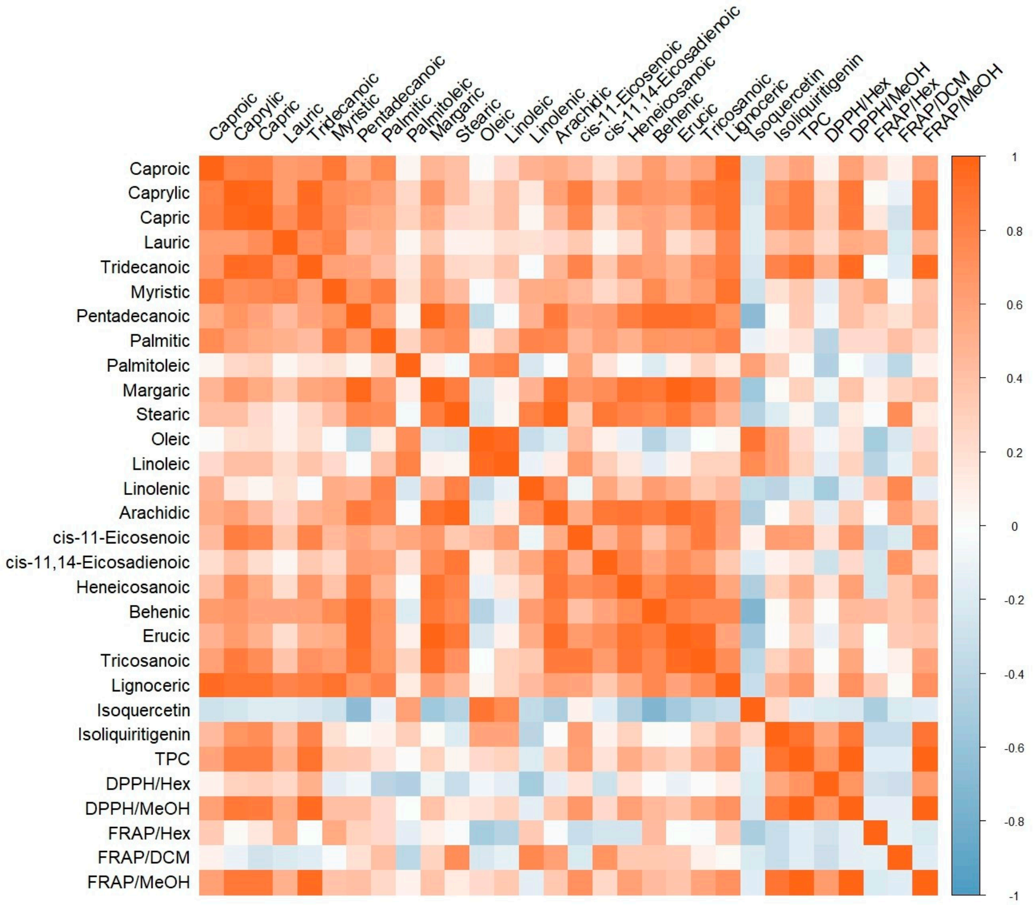

Figure S1. Correlation heatmap depicting the relationships between phytochemical components and antioxidant properties.
